# Stable PbS colloidal quantum dot inks enable blade-coating infrared solar cells

**DOI:** 10.1007/s12200-023-00085-0

**Published:** 2023-10-26

**Authors:** Xinzhao Zhao, Mingyu Li, Tianjun Ma, Jun Yan, Gomaa Mohamed Gomaa Khalaf, Chao Chen, Hsien-Yi Hsu, Haisheng Song, Jiang Tang

**Affiliations:** 1 Wuhan National Laboratory for Optoelectronics (WNLO), Huazhong University of Science and Technology (HUST), Wuhan, 430074 China; 2 School of Optical and Electronic Information, Huazhong University of Science and Technology (HUST), Wuhan, 430074 China; 3 School of Energy and Environment and Department of Materials Science and Engineering, City University of Hong Kong, Hong Kong, 999077 China; 4 Wenzhou Advanced Manufacturing Technology Research Institute of Huazhong University of Science and Technology, Wenzhou, 325035 China

**Keywords:** PbS quantum dots, Solvent engineering, Colloid stability, Blade coating, Infrared solar cells

## Abstract

Infrared solar cells are more effective than normal bandgap solar cells at reducing the spectral loss in the near-infrared region, thus also at broadening the absorption spectra and improving power conversion efficiency. PbS colloidal quantum dots (QDs) with tunable bandgap are ideal infrared photovoltaic materials. However, QD solar cell production suffers from small-area-based spin-coating fabrication methods and unstable QD ink. Herein, the QD ink stability mechanism was fully investigated according to Lewis acid–base theory and colloid stability theory. We further studied a mixed solvent system using dimethylformamide and butylamine, compatible with the scalable manufacture of method-blade coating. Based on the ink system, 100 cm^2^ of uniform and dense near-infrared PbS QDs (~ 0.96 eV) film was successfully prepared by blade coating. The average efficiencies of above absorber-based devices reached 11.14% under AM1.5G illumination, and the 800 nm-filtered efficiency achieved 4.28%. Both were the top values among blade coating method based devices. The newly developed ink showed excellent stability, and the device performance based on the ink stored for 7 h was similar to that of fresh ink. The matched solvent system for stable PbS QD ink represents a crucial step toward large area blade coating photoelectric devices.

**Graphical Abstract:**

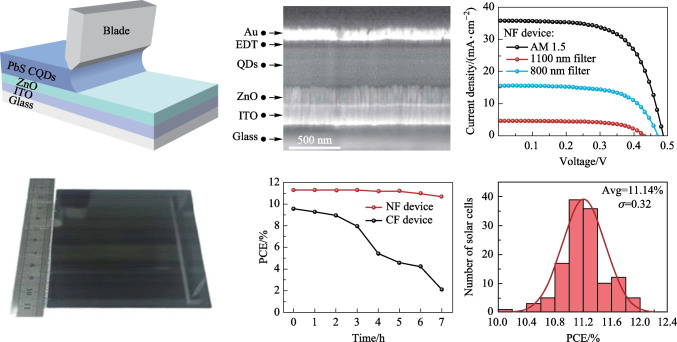

**Supplementary Information:**

The online version contains supplementary material available at 10.1007/s12200-023-00085-0.

## Introduction

Because of the tunable bandgap, solution processability, and modifiable surface [1], quantum dots (QDs) are now widely used in light-emitting diodes [2, 3], solar cells [4], field-effect transistors [5], photodetectors [6], and so on. In the field of solar cells, near-infrared QDs have become an ideal bottom cell absorber for tandem devices due to their quantum size effect, low-cost solution fabrication, and high infrared absorption coefficient [7–12]. The theoretical research shows that the tandem device consisting of a 1.55 eV perovskite top-cell and 1.0 eV PbS QDs bottom-cell can achieve an ideal power conversion efficiency (PCE) of 43%, which is much higher than the 33% single junction limit [13]. In comparison with all perovskite tandem solar cells, such a tandem device also holds special advantages of high working stability and low production cost. Thus, QD near-infrared solar cells (IRSCs) show high potential in next-generation photovoltaics and tandem solar cells.

For thin film solar cell fabrication, it is very important to produce homogeneous, dense, and large-area films. Nowadays, benefiting from the development of the solution-phase ligand exchange method [14], high-quality QD films can be prepared by a single step of spin-coating. Employing a high-speed gas flow can result in rapid drying of the solvent, to obtain a smooth and continuous QD film. We have previously reported the sputtered ZnO-based PbS QD devices by spin-coating (*E*_g_ = 0.97 eV) and realized 3.90% and 10.47% certified PCE under 800 nm filter and AM1.5G conditions, respectively [15]. However, it is worth noting that the spin-coating process always wastes a significant quantity of QD ink, which increases the manufacturing cost greatly. Moreover, spin-coating is a small-area deposition method and is not conducive to the large-scale manufacturing of QD solar cells and this is a major obstacle to commercial development.

Blade coating without centrifugal force is expected to solve these problems and achieve low-cost, large-area preparation of QD films [16]. Sukharevska et al. have demonstrated the preparation of stable QD ink and large-scale film by blade coating with a PCE of 8.7% [17]. The development of QD ink film production from the mature spin coating process to the blade coating process still faces many physical and chemical challenges. The selection of solvents is an important factor in preparing high-quality films by blade coating [16–18]. Specifically, the solvent physical parameters should match the blade coating process. The suitable solvent can prolong the effective window period of film preparation, and ink with a specific viscosity can prevent the autoflow of the wet film to reduce macro defects. At the same time, the solvent needs to have a suitable boiling point to avoid uneven film caused by fast solvent evaporation [17].

Here, we developed a mixed solvent system from dimethylformamide (DMF) and butylamine (BTA) according to Lewis acid–base theory and DLVO theory. This system can maintain the stability of QD inks and is compatible with the blade coating process. The stability time of QD ink based on this system exceeded 7 h, and 100 cm^2^ uniform QD films were successfully obtained by blade coating. The large area QD film based devices achieved an average PCE of 11.14% and an 800 nm-filtered PCE of 4.28%, which are the top values achieved in reported research literature to date for the blade coating method.

## Materials and methods

### Chemicals

Thioacetamide (TAA) (≥ 99%), zinc stearate (≥ 90%), 1-octadecene (ODE) (Aladdin, ≥ 90%), oleylamine (OLA) (Aladdin, 90%), ethanol (Sinopharm, ≥ 99.7%), lead chloride (PbCl_2_) (Aladdin, 99.99%), acetone (Sinopharm, ≥ 99.5%), oleic acid (OA) (Alfa Aesar, ≥ 90%), n-octane (Sinopharm, ≥ 95%), hexylamine (Aladdin, 98%), hexamethyldisilathiane (TMS) (TCI, ≥ 95%), DMF (Aladdin, ≥ 99.8%), BTA (Aladdin, ≥ 98%), indium tin oxide (ITO) (Advanced Election Technology Co. Ltd.), lead bromide (PbBr_2_) (Advanced Election Technology Co. Ltd., ≥ 99.99%), lead iodide (PbI_2_) (Advanced Election Technology Co. Ltd., ≥ 99.999%), ethyl acetate (Sinopharm, ≥ 99.7%), Dimethyl sulfoxide (DMSO) (Aladdin, ≥ 98%), N-Methyl-2-pyrrolidone (NMP) (Aladdin, ≥ 99%), Pyridine (Py) (Aladdin, ≥ 98%), Propylene carbonate (PC) (Aladdin, ≥ 98%), 1, 2-ethanedithiol (EDT) (Aladdin, ≥ 97%) and acetonitrile (Sinopharm, ≥ 99.8%).

### Synthesis of small-size PbS QDs (exciton peak ~ 880 nm)

Lead oxide, oleic acid, and ODE were placed in a 250 mL three-necked flask and heated under a negative pressure condition for 1.5 h, to obtain lead oleate solution. Then, under intense agitation, the TMS was injected into the lead oleate solution. One minute later, the three-necked flask was bathed to room temperature to generate small-size quantum dots. PbS QDs purified with ethyl acetate and ethanol were finally dispersed in n-octane (40 mg/mL) for EDT layer deposition.

### Synthesis of ZnS QDs (absorption peak ≈ 256 nm)

3.6 g thioacetamide (0.048 mol), 70.56 g zinc stearate (0.096 mol), 221 g ODE and 156 g OLA were put into a 2 L three-neck flask. The mixture was heated to 140 °C under a nitrogen atmosphere and maintained for 50 min. Then, the water bath, was cooled to 40 ℃ and 32 mL n-octylamine was added. Finally, n-hexane and ethanol were used as antisolvents to precipitate ZnS QDs [27].

### Synthesis of large-size PbS QDs (exciton peak ≈ 1290 nm)

1.946 g PbCl_2_ (0.007 mol) and 18.94 g OLA were placed into a 250 mL three-mouth flask. The mixture was heated to 140 ℃ under a nitrogen atmosphere and maintained for 30 min. Then the flask was cooled to 60 °C and ZnS QDs were rapidly injected for nucleation. After that, ZnS QDs were slowly injected for growth (absorbance was 0.3 after 3000 times dilution). The growth temperature gradually increased from 60 °C to 100 °C, and the whole growth process lasted for about 90 min. Then, the temperature was cooled to 70 °C by water bath, 90 mL hexane was injected, and 40 mL oleic acid was injected at 40 °C. Finally, the mixture was purified with acetone to obtain solid PbS QDs [28].

### Preparation of solar cells

Firstly, the ZnO layer was deposited by sputtering on a glass-ITO substrate. Secondly, the absorption layer was prepared by scraping. Specifically, the ligand solution of PbI_2_:PbBr_2_ was prepared with 10 mL DMF, and the molar ratio of PbI_2_ to PbBr_2_ was 9.2:1. An equal volume of 10 mg/mL QDs solution in n-octane was then prepared. The above two were thoroughly mixed for ligand exchange, and the exchanged solid QDs were obtained by centrifugation. Then 700 mg/mL QD ink was prepared with two mixed solvents (BTA:DMF = 4:1 and BTA:DMF = 3:7) for blade coating. The QD films were deposited by blade coating on the ZnO substrate at 40 °C. The width of the slit was 90 μm and the coating speed was 10 mm/s. The wet film was annealed at 90 ℃ for 10 min to obtain a dry QD film with a thickness of about 350 nm. The whole process was carried out under a nitrogen atmosphere. Thirdly, PbS QD film (exciton peak ~ 880 nm) was treated with EDT ligand used as hole transport layers. Finally, 80 nm gold was deposited on the top as the top electrode. The effective area of the solar cell was 0.04 cm^2^.

### Materials and device characterizations

The optical absorption spectra of the QDs were measured by a Shimadzu UV-3600 Plus spectrophotometer. Photoluminescence (PL) spectra of QDs were measured using HORIBA modular scientific research grade fluorescence spectrometer. The QD film surface topography was measured by atomic force microscope (AFM) SPM9700. The scanning electron microscope (SEM) images were obtained by FEI Nova Nano SEM 450. The PbS QD film crystallization was tested by X-Ray diffraction (XRD) with Cu K*α* radiation (Philips, X pert pro-MRD, Netherlands). The current density–voltage (*J*–*V*) curve was obtained under a simulated AM 1.5 (100 mW/cm^2^) solar spectrum from a 450 W xenon lamp (Oriel, Model 9119, Newport). 800 (Thorlabs FELH-0800) and 1100 nm (Thorlabs FELH-1100) long pass filters were used to simulate the four-terminal tandem configurations with perovskite and silicon, respectively. Capacitance–voltage (*C*–*V*) measurements were conducted using an Agilent 4200A at a frequency of 100 kHz and an AC signal of 50 mV, scanning from − 0.7 to 0.7 V, in steps of of 20 mV. The drive-level capacitance profiling (DLCP) measurement of the devices was performed with variant amplitude (≈ − 0.7–0.6 V) and frequency (10–500 kHz). The transient photocurrent (TPC) and transient photovoltage (TPV) measurements were performed on the device under dark conditions. A ring of red light-emitting pulse diode (LED, Lumiled) was controlled by a fast solid-state switch, and the pulse width was 1 ms. The TPC was measured using 40 Ω external series resistance to operate the device in short-circuit conditions. Similarly, TPV was applied using 1 MΩ external series resistance to operate the device in open-circuit conditions. Images of QD aggregates were obtained using a transmission electron microscope (TEM) instrument (JEOL 100FJEM-2100F). The external quantum efficiency of the solar cell was obtained using a McScience K3100IQX measurement system (100 Hz chopper monochromatic illumination).

## Results and discussion

In order to make QD ink compatible with blade coating, the ink solvent recipe was studied. The stability of PbS-IBr (PbI_2_ and PbBr_2_ capped PbS QDs) ink depends on the electrostatic interaction between the charged surface of QDs and the surrounding solvent. The PbS (111) facet is lead-rich [19], for dissolving the QDs, solvents need to have sufficient capacity to provide lone pair electrons; that is, sufficient Lewis basicity can help to dissociate ligand precursors. To study the exact effect of solvent on the ink, I/Br capped PbS QDs were dissolved in typical basic solvents; there were BTA, NMP, PC, Py, etc. As shown in Fig. S1a, QDs only dispersed in BTA due to its strong Lewis basicity. Key physical parameters for the above solvents are listed in Table S1. However, BTA does not maintain the stability of the ink. As shown in Fig. S1b, for BTA-based ink, no obvious exciton peak of QDs was obtained after 4 h of storage, indicating the degradation of QDs.

**Fig. 1:**
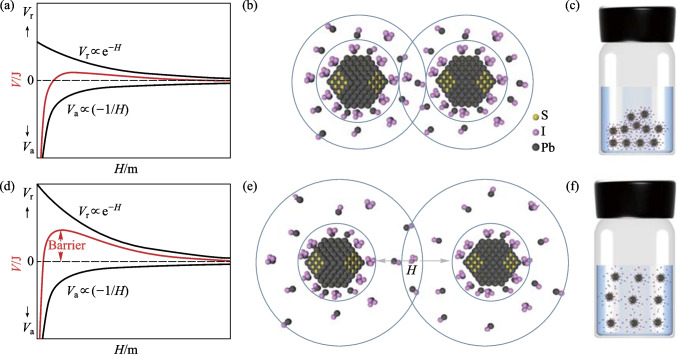
Schematic diagram of potential energy, electrostatic double layer structure, and colloidal particle dispersion of quantum dots dispersed in a low dielectric constant solvent (**a**–**c**) and high dielectric constant solvent (**d**–**f**)

The instability of BTA-based ink was analyzed by DLVO theory. (Details of the theory are given in the supporting information [20], Additional file 1.) Fig. 1a shows the relationship between the potential energy of colloidal particles and spatial spacing. And the potential energy (*V*) is the sum of gravitational potential energy (*V*_a_) and repulsive potential energy (*V*_r_). When the dielectric constant of the solvent is small, the corresponding *V* is very small, and has difficulty in impeding QD aggregation. As shown in Fig. 1b, c, there is a serious overlap in the diffusion layer of the colloidal particles, resulting in colloidal instability. On the contrary, when the dielectric constant of the solvent is large, as shown in Fig. 1d, there is a much higher potential energy compared with the scenario of Fig. 1a. As shown in Fig. 1e, f, high potential energy can keep a low degree of overlap in the diffusion layer and maintain the stability of the colloid. Thus, the lowest dielectric constant of BTA ~ 4.9 in our investigation was not sufficient to maintain the stability of the ink. Moreover, the boiling point of BTA at standard atmospheric pressure is only 78 ℃, which makes it evaporate rapidly at room temperature and it is incompatible with the large-area preparation process [21, 22]. On the other hand, solvents with boiling points that are too high are difficult to remove in subsequent annealing processes. Therefore, an optimal solvent should have strong Lewis basicity, a high dielectric constant, and a suitable boiling point. It is difficult for a single solvent to meet all of the above requirements, thus we adopted a mixed solvent strategy to address this challenge.

The strong Lewis basicity of BTA makes it one of the essential components of mixed solvents. A series of solvents was investigated to cooperate with BTA in a volume ratio (*V*:*V*) of 1:1. QDs can be fully dispersed in all kinds of mixed solvents, as shown in Fig. S1a. After the ink was stored for 4 h, the QDs were coagulated in low dielectric constant solvents such as Py and BTA, while the solvents with high dielectric constant ones (DMF, NMP, DMSO, PC) exhibited higher QD stability. Absorption spectra of different inks are shown in Fig. S1b. Solvents with high dielectric constants such as PC, DMSO, and DMF show more obvious exciton absorption peaks. It should also be noted that high dielectric constant solvents are accompanied by high boiling points, which brings new difficulties for subsequent film deposition (Table S1). Finally, a mixed solvent of BTA and DMF with a suitable boiling point was selected for blade coating ink preparation.

**Fig. 2:**
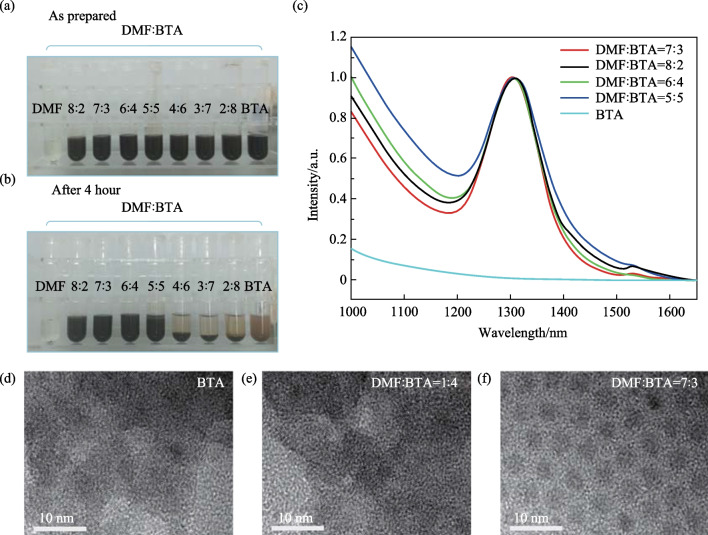
**a**, **b** Photos of quantum dot solutions dispersed in different proportions of mixed solvent (DMF + BTA). **c** Absorption spectra of different inks after 4 h of storage. **d**–**f** TEM images of different inks after 4 h of storage, with solvent ratios of BTA only, DMF: BTA = 1:4, and DMF: BTA = 7:3, respectively

The optimal proportion of DMF: BTA was further investigated. As shown in Fig. 2a, b, and S2, after 4 h of storage, the ink with DMF: BTA smaller than 4:6 ratio showed significant phase separation, while at a ratio over 1:1 there was good dispersion. The relevant exciton absorption peaks are shown in Fig. 2c. The 7:3 ratio of ink demonstrated more perfect absorption spectra; the detailed parameters of different inks are shown in Table S2. To obtain the microstructure of QDs after 4 h of storage, typical ink samples were characterized by TEM (Fig. 2d–f). The ink prepared by pure BTA showed large aggregates with no QD morphologies (Fig. 2d). The inks (1:4) displayed similar aggregates. This indicated that a low proportion of DMF could not provide a suitable aggregated barrier to maintain colloid stability (Fig. 2e). In contrast, as shown in Fig. 2f, ink with a higher proportion of DMF (7:3) showed mono-dispersed distribution. TEM image results agreed well with their absorption spectra (Fig. 2c), and a 7:3 proportion was utilized for blade coating thereafter.

**Fig. 3:**
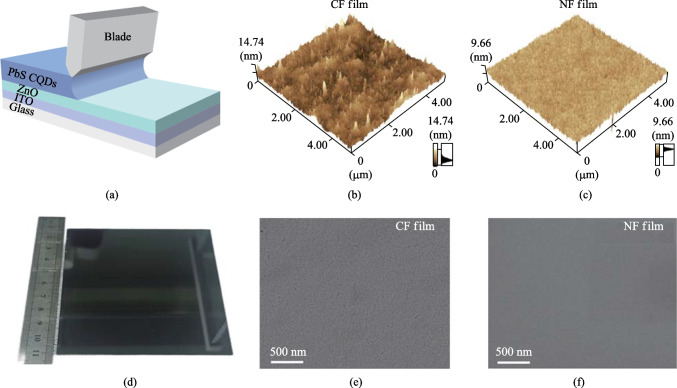
**a** Schematic diagram of blade coating. **b**, **c** The AFM images of CF film and NF film. **d** Photo of a blade-coated film by the NF. **e**, **f** The SEM images of CF film and NF film

Two kinds of inks based on the new formula (NF) of DMF: BTA = 7:3 and the control formula (CF) with DMF: BTA = 1:4 were utilized for blade coating and device fabrication. Figure 3a schematically shows the blade coating process for QD film. To evaluate the morphology of the films at the nanoscale, the QD films of NF and CF were characterized by AFM and SEM. As shown in Fig. 3b, c, the average roughness of the CF film was 1.3 nm, while the corresponding value of NF film was only 0.6 nm. From the AFM image comparison, the CF film demonstrated distinct surface morphologies rich in spike-like peaks and deep concaves. On the contrary, the NF film image shows a much smoother surface with uniform contrast. A higher proportion of DMF can increase the dynamic viscosity of ink and effectively alleviate the QD ink flow resulting in a smoother film. Ascribing to the NF-based ink, a QD film ~ 100 cm^2^ was successfully deposited as shown in Fig. 3d. The whole film surface demonstrated high uniformity and density. Figure 3e and f show their SEM images. The CF film had a large number of holes and cracks, while the NF film exhibited a more compact and smooth appearance. Compared with NF, the faster rate of CF volatilization led to stress related holes and cracks increasing the roughness of the film. Figure S3a shows XRD spectra that characterize the crystallization of QD films. The XRD half-width at half-maximum (HWHM) values of the CF and NF films were wide due to the small grain size of the QD film.

**Fig. 4:**
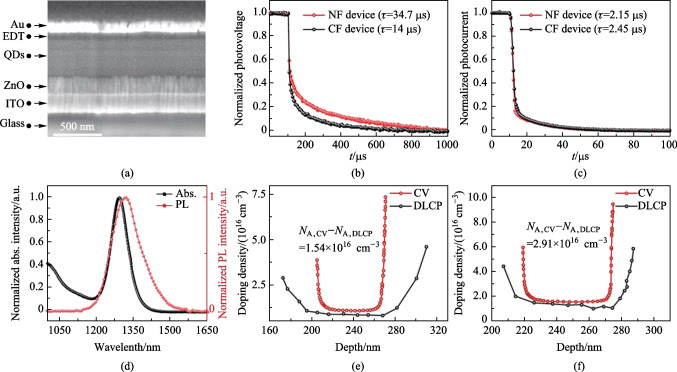
**a** SEM image of device cross-section. **b**, **c** Normalized TPV and TPC decay curves. **d** Absorption and PL curve of QDs. **e**, **f** CV and DLCP curves of the CF device and the NF device respectively

To evaluate the influence of the above two kinds of films on the device performance, we prepared two batches of devices in classic device structure of Glass/ITO/ZnO/PbS-IBr/PbS-EDT/Au [23]. Figure 4a shows the device cross-section SEM image, each layer demonstrates a clear interface with conformal deposition. Figure 4d shows the absorption and PL spectra of QDs. The peaks are located at 1290 and 1313 nm, respectively. The Stokes shift is 23 nm, and the peak-to-valley ratio of the absorption spectrum is 9.2. Compared with literature data, the higher peak-to-valley ratio and the smaller Stokes shift indicate the high quality and monodispersity of quantum dots[15]. To study the recombination and extraction dynamics of the devices, their TPV and TPC curves were measured and are shown in Fig. 4b, c, respectively. From the results of the TPV measurement, the carrier lifetime of the NF device was shown to be 34.74 μs, which is much longer than that of the CF device (14 μs). The longer carrier lifetime indicated that the deep defects in the film were suppressed [24]. For TPC spectra, the NF device obtained a faster charge extraction rate compared with the CF device. Transient dynamics of IRSCs indicated that the choice of solvent affected the dynamics of recombination and extraction. To further analyze the PN junction interface defect density, we characterized *C − V* and DLCP for CF and NF devices [25]. As shown in Fig. 4e and f, the interface defect density of the NF device is 1.54 × 10^16^ cm^−3^, significantly lower than that of CF devices (2.91 × 10^16^ cm^−3^). Combined with above characterizations of QD films and devices, a reliable conclusion can be reached that the higher viscosity and moderate boiling point of NF ink are more compatible with the blade coating process.

**Fig. 5:**
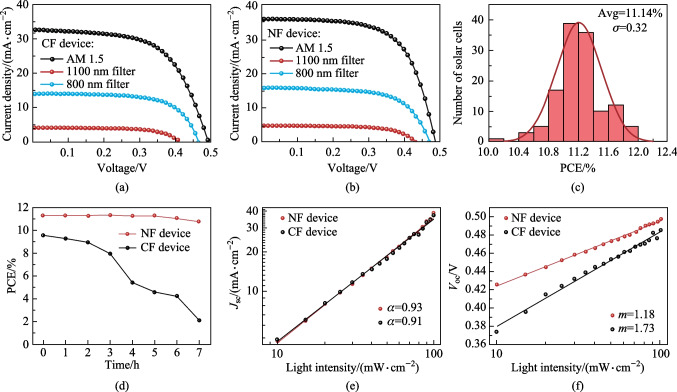
**a**, **b** *J − V* curves of CF device and NF device. **c** Statistical histogram of PCE of the NF device. **d** PCE curve of the device prepared with ink and stored for different times. **e**, **f** Light intensity dependence of *J*_sc_ and *V*_oc_ of the two devices

The *J − V* characteristic curves of IRSCs are shown in Fig. 5a, b. The average PCE of CF devices was 9.56%. On the contrary, the average values of the NF devices reached 11.14% under AM1.5G illumination, 4.28% after 800 nm filtering, and 1.28% after 1100 nm filtering. All PCE parameters of NF device were the top values among blade coating devices (Table S3). Detailed performance parameters of devices under different lighting conditions are listed in Table 1. The key device parameters, *J*_sc_ and FF, were improved from 32.55 mA/cm^2^ and 59.62% to 35.71 mA/cm^2^ and 64.11%, due to the lower interface defect density of NF films. Fig. S3b shows the external quantum efficiency and integrated current of NF and CF devices. The integrated currents of the full spectrum were 35.69 mA/cm^2^, for NF, and 32.45 mA/cm^2^, for CF, following a similar pattern to that in the *J*_sc_ data in the *J − V* curve. To investigate the uniformity of NF films, 128 operating points were randomly selected from 100 cm^2^ QDs film (Fig. 3d). Figure 5c shows the PCE statistical histogram. It obtained an average conversion efficiency of 11.14% and a standard deviation of 0.32%. Such a small standard deviation reflected the excellent uniformity of the blade coating active absorber. We measured the maximum power point tracking (MPPT) and storage stability of the champion device. As shown in Fig. S4a, the device exhibited a stable power output under continuous operation at the maximum power point (MPP) (with AM1.5G illumination lasting 1000 s). At the same time, the device showed excellent storage stability. As shown in Fig. S4b, the PCE decreased from 11.13% to 10.64% after storage for 1000 h under nitrogen atmosphere. To observe the stability of the ink, we monitored the PCE of devices prepared by ink with different storage times, as shown in Fig. 5d. After 3 h of storage, the PCE of the CF device began to decrease significantly, from 9.58% to 8%. On the contrary, the NF-based PCE after 7 h of storage was similar to the fresh ink based values, which indicated that NF inks could prolong the effective window period for film preparation. The defect recombination mechanism of IRSCs can be analyzed by light intensity (*I*) dependence of *V*_oc_ and *J*_sc_. The *J*_sc_ ∝ *I*^*α*^ curves in double-logarithmic scale are shown in Fig. 5e. The slopes (*α*) of the NF and CF devices are 0.93 and 0.91, respectively. It suggests that non-geminate recombination in CF cells is stronger than that in NF cells [26]. The *V*_oc_ − *I* relationship satisfies *δV*_oc_ = *mkT*/*q* (Fig. 5f), where *kT* is the thermal energy, *q* is the elementary charge, and *m* is the diode ideality factor. The *m* value of NF IRSCs was 1.18. a decrease from 1.73 of the control device, which implies that the nonradiative combination dominated by traps was restrained significantly in the former IRSCs due to superior QD ink quality and its related absorber film.

**Table 1:** Average PCE of two kinds of solar cells

Device	Solar illumination	*V*_oc_/V	*J*_sc_/(mA⋅cm^−2^)	FF/%	PCE/%
CF	AM 1.5	0.49	32.55	59.62	9.58
	800 nm filter	0.47	14.13	61.93	4.09
	1100 nm filter	0.42	4.06	62.94	1.06
NF	AM 1.5	0.49	35.71	64.11	11.14
	800 nm filter	0.47	15.64	64.51	4.73
	1100 nm filter	0.43	4.60	64.69	1.28

In terms of the aforementioned discussion, we can conclude that the photovoltaic performance enhancement of IRSCs with NR solvent engineering originated from the effectively suppressed non-geminate and nonradiative recombination.

## Conclusions

In summary, based on Lewis acid–base theory and DLVO theory, we developed a stable QD ink compatible with the blade coating process and achieved QD IRSCs with high efficiency and scalable manufacturing. The contact barrier between QDs was changed by adjusting the polarity of the mixed solvent. Finally, the stability time of QD ink based on NF exceeded 7 h, and a uniform, dense, and low surface defect density QD film of 100 cm^2^ was obtained by blade coating. We selected 128 working points from a 100 cm^2^ QD film. The average PCE was 11.14% with a small standard deviation of 0.32% and the 800 nm-filtered average PCE was 4.28%, all of which, according to the published literature, are the highest values achieved to date for blade coating PbS QD solar cells. Further device physics demonstrated suppressed charge recombination and rapid charge extraction. The drive-level capacitance profiling analysis showed that the compact NF film had a lower interface defect density. The optimal solvent system by Lewis acid–base theory and DLVO theory could provide a reference for the stabilized QD ink. The developed solvent system for obtaining uniform and large area QD film represents a crucial step toward commercial viability of PbS QD photovoltaics.

### Supplementary Information

**Additional file 1**. Supporting Information.
