# Supplementary material for: Stable PbS colloidal quantum dot inks enable blade-coating infrared solar cells
Source: Front Optoelectron. 2023 Oct 26;16(1):27. doi: 10.1007/s12200-023-00085-0 (PMC10602987; doi:10.1007/s12200-023-00085-0)
Supplement: Supplementary file 1 — Additional file 1. Supporting Information. [file 12200_2023_85_MOESM1_ESM.pdf]

Supporting Information

Stable PbS colloidal quantum dot inks enable blade-coating infrared solar cells

Xinzhao Zhao[1+], Mingyu Li[1+], Tianjun Ma[1], Jun Yan[1], Gomaa Mohamed Gomaa Khalaf[1], Chao Chen[2], Hsien-Yi Hsu[3*], Haisheng Song[1, 2, 4*], Jiang Tang[1, 2, 4]

[1]Wuhan National Laboratory for Optoelectronics (WNLO), Huazhong University of Science and Technology (HUST), 1037 Luoyu Road, Wuhan 430074, Hubei, P. R. China

[2] School of Optical and Electronic Information, Huazhong University of Science and Technology (HUST), 1037 Luoyu Road, Wuhan 430074, Hubei, P. R. China

[3]School of Energy and Environment & Department of Materials Science and Engineering, City University of Hong Kong, Kowloon Tong, Hong Kong 999077, People's Republic of China

[4] Wenzhou Advanced Manufacturing Technology Research Institute of Huazhong University of Science and Technology, Wenzhou, Zhejiang, P. R. China

[+]These authors contributed equally to this work.

[*] Correspondence email: songhs-wnlo@mail.hust.edu.cn (H. Song)

1. DLVO theory

According to the DLVO theory, there are two independent forces between particles in the liquid. The first is the van der Waals gravitation, which causes the particles to coagulate. The second is the electrostatic repulsion caused by the overlap of the diffused electrostatic double layer, which is the reason for maintaining colloid stability. The stability of colloids depends on the larger one.

Firstly, according to Hamaker's hypothesis, the gravitational potential energy resulting from van der Waals attraction between colloidal particles is expressed as

$$V_a = -\frac{Ar}{12H}, \tag{1}$$

where r is the particle radius in m, H is the shortest distance from the particle surface in m, and A is the Hamaker constant in J.

Secondly, colloidal particles have an electric double-layer structure. Once the ligand on the QDs surface is dissociated in a strong Lewis basicity environment, most of the PbI_3 ions will attach to the charged surface of the QDs, forming a "stern" layer, which together with the QDs forms colloidal particles; Most PbI^+ ions will form a

diffusion layer around colloidal particles due to electrostatic interaction. The electric double-layer is composed of colloidal particles and their diffusion layer is electrically neutral, so the diffusion layer acts as a shielding layer to shield charged particles from electrostatic repulsion. However, when colloidal particles are close to each other, the diffusion layer will overlap. Overlapping will destroy the charge distribution of the diffusion layer and will affect the electrostatic balance and potential of the electric double-layer, thus damaging the shielding of colloidal particles and generating electrostatic repulsion between each other. The expression of electrostatic repulsion potential energy is

$$V_r = \frac{64\pi r n_0 kT {\gamma_0}^2}{\kappa^2} \exp(-\kappa H)\,, \tag{2}$$

where *r* is the radius of colloidal particles in m, *H* is the shortest distance between particle surfaces in m, N_0 is the number density of positive or negative ions in the solution, *K* is the Boltzmann constant, and *T* is temperature. The definition formula of γ_0 is

$$\gamma_0 = \frac{\exp(Ze\psi_0/2kT) - 1}{\exp(Ze\psi_0/2kT) + 1}, \tag{3}$$

where ψ_0 is the surface potential of colloidal particles, *Z* is the ionic valence state, and *e* is the electric quantity of the electron.

In Eq. (2), κ^{-1} is the thickness of the electric double-layer, and its definition formula is

$$\kappa = \left(\frac{2Z^2 e^2 n_0}{\varepsilon kT}\right)^{1/2}, \tag{4}$$

where ε is the dielectric constant of the solvent.

According to Eqs. (2), (3), and (4), the electrostatic repulsive potential energy is not only related to the geometric factors *r* and *H* between colloidal particles but also depends on the surface potential, electrolyte concentration, ionic valence state, and solvent dielectric constant.

The total potential energy is the sum of gravitational potential energy and repulsive potential energy.

$$V = V_a + V_r. \tag{5}$$

2. Figures and tables

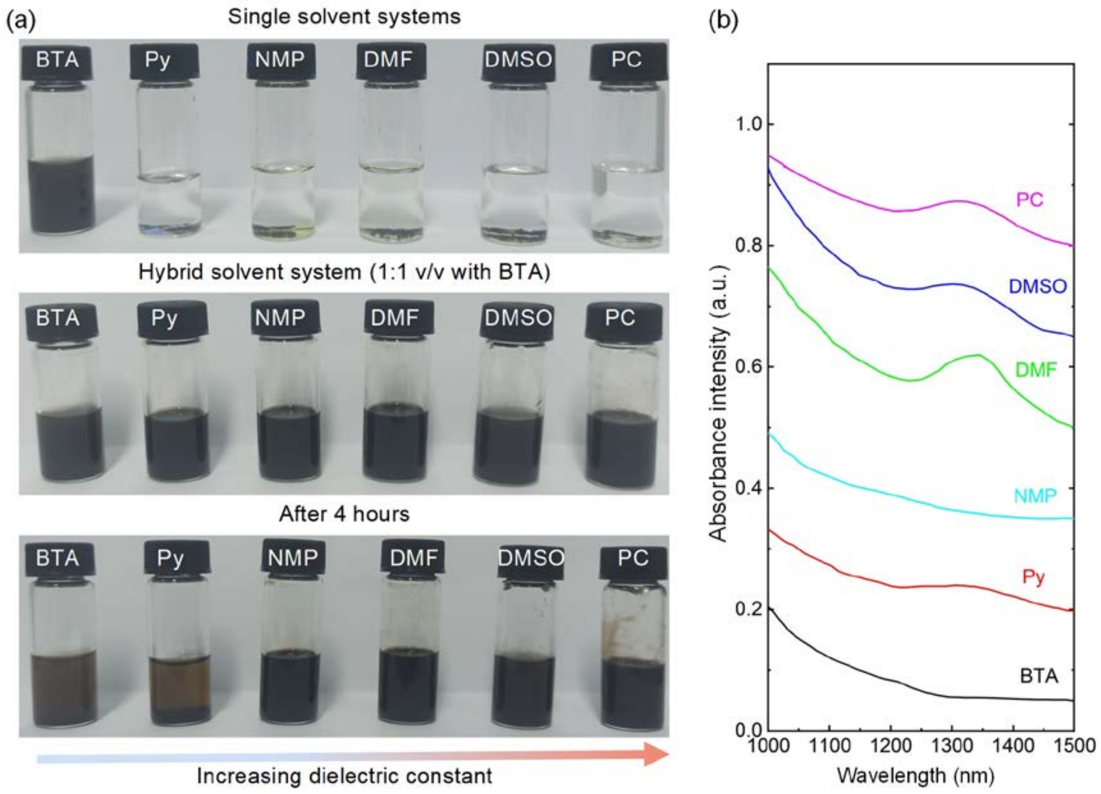

Figure S1. a) Photos of quantum dots dispersed in different solvents. b) The absorption spectra of quantum dots dispersed in a mixed solvent system after 4 hours of storage.

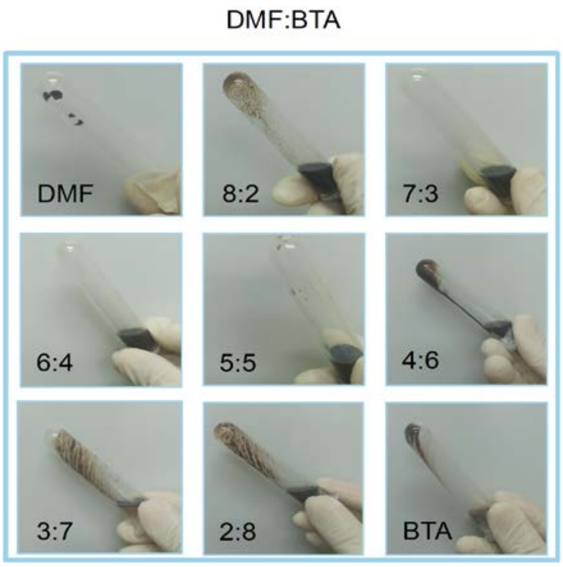

Figure S2. Photos of the bottom of QD ink after 4 hours of storage.

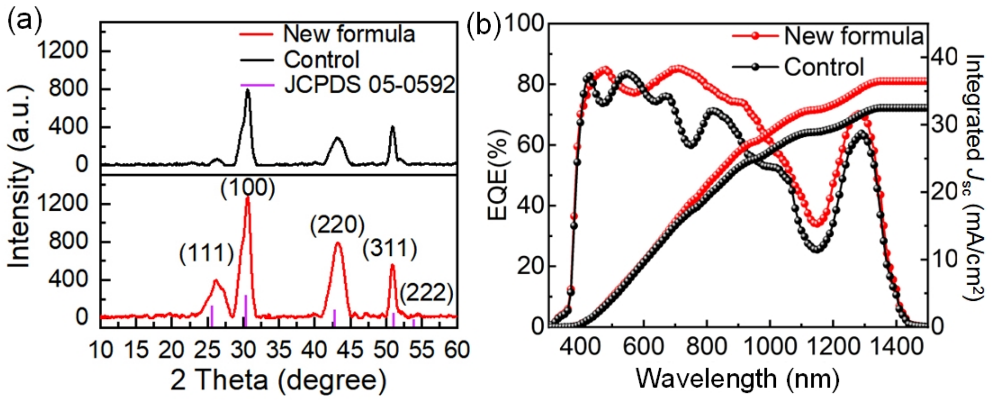

Figure S3. a) XRD diagram of quantum dot film b) EQE and integral current curves of two devices

(a) (b)

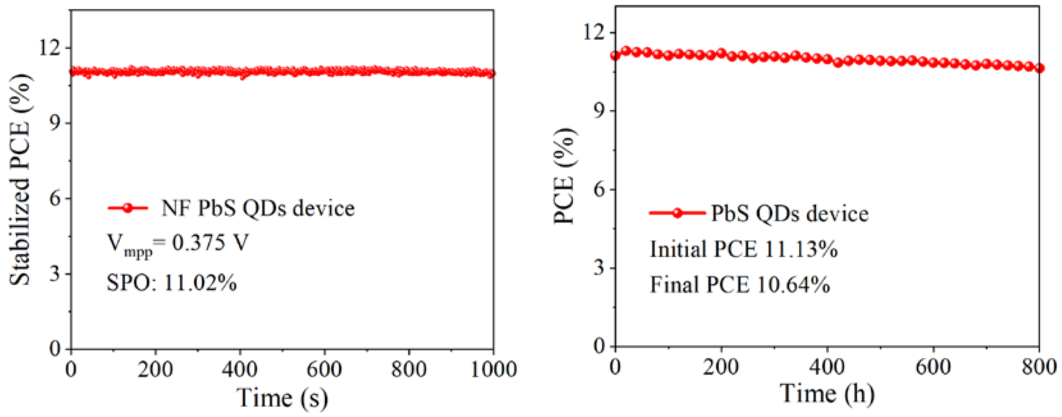

Figure S4. a) The MPPT the champion device, SPO: Stabilized Power Output. b) The PCE measurement of the champion device stored in the nitrogen atmosphere.

Table S1. Chemical parameters of different solvents

Solvent	Dielectric constant [a.u.]	E_T^N [a.u.]	D_N [Kcal /mol]	BP [°C]
n-Butylamine (BTA)	4.9	0.21	42	78
Propylene carbonate (PC)	64	0.472	15.1	242
Dimethyl formamide (DMF)	36	0.386	26.6	153
1-Methyl-2-pyrrolidinone (NMP)	33	0.355	27.3	202
Dimethyl sulfoxide (DMSO)	48	0.444	29.8	189
Pyridine (Py)	12	0.302	33.0	115

Table S2. Physical parameters corresponding to the absorption curves of Figure 2c

DMF:BTA	DMF	8:2	7:3	6:4	5:5	4:6	3:7	2:8	BTA
first excitonic peak(nm)	N / A	1308	1304	1306	1306	N/A	N/A	N/A	N/A
peak-to-valley ratio	N / A	2.60	3.05	2.5	1.94	N/A	N/A	N/A	N/A

Table S3. Summary of previously reported QDs IRSC and our work. N/A refers to non-reported values.

Device	absorption peak of QDs	Solar illumination	V_{oc} [V]	J_{sc} [mA/cm^2]	FF [%]	PCE [%]
This work	0.96 eV	AM 1.5	0.49	35.71	64.11	11.14
		800 nm filter	0.47	15.64	64.51	4.73
[1]	1.34 eV	AM 1.5	0.57	25.0	61.00	8.7
		800 nm filter	N/A			
[2]	1.32 eV	AM 1.5	0.59	27.75	0.62	10.01
		800 nm filter	N/A			
[3]	1.42 eV	AM 1.5	0.62	25.31	68.01	10.67
		800 nm filter	N/A			
[4]	0.75 eV	AM 1.5	0.35	38.9	42.8	5.74
		800 nm filter	N/A			
[5]	1.32 eV	AM 1.5	0.57	26.22	0.61	9.04
		800 nm filter	N/A			
[6]	0.96 eV	AM 1.5	0.47	35.46	59.5	10.0
		800 nm filter	0.44	15.77	61.2	4.2

References:

[1] Sukharevska, N., Bederak, D., Goossens, V. M., Momand, J., Duim, H.: Scalable PbS Quantum Dot Solar Cell Production by Blade Coating from Stable Inks. ACS Appl. Mater. Interfaces **13**(4), 5195-5207 (2021)

[2] Aqoma, H., Jang, S. Y.: Solid-state-ligand-exchange free quantum dot ink-based solar cells with an efficiency of 10.9%. Energy Environ. Sci. **11**(6), 1603-1609 (2018)

[3] Lu, K., Meng, X., Liu, Z., et al.: Packing state management to realize dense and semiconducting lead sulfide nanocrystals film via a single-step deposition. Cell Phys. Sci. **1**(9), (2020)

[4] Fan, J. Z., Vafaie, M., Bertens, K., Sytnyk, M., Pina, J. M.: Micron Thick Colloidal Quantum Dot Solids. Nano Lett. **20**(7), 5284-5291 (2020)

[5] Goossens, V. M., Sukharevska, N. V., Dirin, D. N., et al.: Scalable fabrication of efficient pn junction lead sulfide quantum dot solar cells. Cell Phys. Sci. **2**(12), (2021)

[6] Xia, Y., Liu, S., Wang, K., et al.: Cation-exchange synthesis of highly monodisperse PbS quantum dots from ZnS nanorods for efficient infrared solar cells. Adv. Funct. Mater. **30**(4), 1907379 (2020)
